# Sexually transmitted disease among street dwellers in southern Ethiopia: a mixed methods study design

**DOI:** 10.1186/s12889-020-08584-x

**Published:** 2020-04-03

**Authors:** Negash Wakgari, Terefe Woyo, Emnet Kebede, Hirut Gemeda, Samson Gebremedhin

**Affiliations:** 1 Department of Midwifery, Ambo University, Ambo, Ethiopia; 2 Departments of Midwifery, Hawassa University, Hawassa, Ethiopia; 3 School of Public Health, Addis Ababa University, Addis Ababa, Ethiopia

**Keywords:** Sexually transmitted diseases, Street dwellers, Health seeking behavior, Southern Ethiopia

## Abstract

**Background:**

Lack of knowledge about sexual violence, its consequences, substance use and homelessness are major problems that make street dwellers susceptible to sexually transmitted diseases. Hence, this study assessed knowledge, attitudes and treatment-seeking behaviors related to sexually transmitted diseases among street dwellers in southern Ethiopia.

**Methods:**

An explanatory sequential mixed-methods study design was conducted among 842 respondents. A simple random sampling technique was used to select seven cities among fourteen major cities of the region. The sample was allocated proportionally to each selected city. In order to identify and fill in the required sample size, a snowball sampling technique was used. A pre-tested and structured interviewer-administered questionnaire was used to collect quantitative data. The collected data were entered using Epidata and exported to SPSS version 23.0 for further analysis. Unstructured questionnaires were also used to collect 21 in-depth interviews and 10 key informants’ interviews. Respondents for in-depth interviews were selected purposively during quantitative data collection.

**Results:**

Most street dwellers were aware of (86.7%) and had a favourable attitude towards (84.4%) prevention and management of sexually transmitted diseases. A portion of respondents experienced bad-smelling genital discharge (13.8%), genital ulcers (11.2%) and a burning sensation (14.5%) during urination, in the previous year. Among those who experienced symptoms of sexually transmitted disease, only 15.3% of them received treatment from a health care provider. Fear of questions raised by providers was one of the reasons for not seeking care according to our qualitative findings.

**Conclusions:**

In this study, a significant number of street dwellers reported experiencing symptoms of a sexually transmitted disease. Despite having awareness about sexually transmitted diseases, seeking treatment from a health center was found to be low based on both quantitative and qualitative findings. We recommend that health care providers should undergo special training to address the sexual and reproductive health problems of street dwellers.

## Background

Street dwellers are exposed to harmful practices such as substance abuse, gambling and engaging in risk sexual activities including commercial sex work [1, 2]. Living on the street without any family support and protection make street people more vulnerable to a range of sexual and reproductive health problems such as sexual violence, unwanted pregnancy and sexually transmitted disease (STD) [3–6]. Moreover, homelessness facilitates HIV transmission and its progression due to lack of entertainment, poor nutrition and co-infections with other STDs [7]. Lack of entertainment might enhance HIV transmission and its progression due to lack of other forms of entertainment; some people resort to sex [7]. Likewise, substance use, exchanging sex for money and risky sexual behavior among street dwellers may increase the likelihood of STDs [8]. Perceived discrimination by health care providers, prior bad experiences with health care provision, fears about confidentiality and privacy of health status, and accessibility to current treatments for STDs are some factors that influence access to prevention and treatment for the homeless population [9–11].

The number of street dwellers in Ethiopia is rapidly increasing, particularly in major cities [12, 13]. What is certainly known is that their numbers are increasing for various reasons, including the global population growth, poverty, rapid urbanization, and the AIDS pandemic [13, 14]. Street dwellers are considered one of the vulnerable groups whose survival and developmental progress is jeopardized and who are in need of alternative health care services [15]. For younger people, their vulnerability is aggravated because of a lack of understanding of the changes associated with adolescence. Life skills training could aid them to be self-confident about their choices of sexual activities and empower them either to resist sex or have safer sex [16, 17]. Moreover, life skills such as critical thinking can assist them in making sound decisions about when and how to use a condom for safer sex [17].

The sexual health problems of street dwellers such as STDs are not the focus of many interventions due to stigma and discrimination by stakeholders and service providers. Homelessness is a unique challenge for street dwellers in terms of getting information about STDs and testing for sexually transmitted infections [9]. Therefore, provision of sexual health care is an area of concern which scientific investigation is lacking between sexes [2, 12]. In Ethiopia despite the ever-increasing number of street people [12–14], limited health and social services and programs are available to address their particular sexual and reproductive health needs. Further limited studies assessed their awareness, perception and health seeking behavior towards STDs. As a result, the purpose of this study was to assess the knowledge, attitudes and treatment-seeking behaviors among street dwellers about STDs in Southern Ethiopia.

## Methods

### Study design, setting and population

An explanatory sequential mixed-method design was conducted among street dwellers in the major towns of Southern Nation, Nationality and People Regional State (SNNPRS), Ethiopia. SNNPRS is one of the nine federal states of Ethiopia and the third largest region with a population of about 19 million. Hawassa is the capital city of the state with an estimated number of 328, 875 inhabitants and a rapidly growing number of street children and women [14]. In addition to Hawassa; Arba Minch, Soddo, Dilla, Hosanna, Durame, Jinka, Werabe, Welkite, Yergalem, Bench, Tercha, Masha and Mizan Aman are also major cities in the region. Regarding the study population, all street dwellers who were within the reproductive age groups [15–49 years] in the state were considered the source population. All street dwellers in the identified reproductive age groups in the selected cities were considered the study population. Street dwellers that were critically sick and unable to respond during the study period were excluded from the study.

### Sample size and sampling procedure

The sample size of the study (*n* = 845) was calculated using the following single population proportion formula: n = (z _(α/2)_)^2^p(1-p)/d^2^, assuming 95% confidence level (z) and 5% margin of error (d), design effect of 2 and 10% compensation for possible non-response. The expected prevalence of the outcomes of interest (p) was set at 50% because we were not able to find reasonable estimates from previous literature.

Our overall sampling approach was a multistage sampling technique to identify the study respondents. Initially simple random sampling technique was employed to select seven cities (Arba Minch, Dilla, Durame, Hawassa, Mizan Aman, Soddo, and Yergalem) among fourteen major cities in the region. A preliminary survey was conducted to determine an estimated number of street dwellers of reproductive age and the sites they frequently used for survival work including mosques, churches, bus stations, open markets, main roads and side street. Moreover, the time when they avail themselves more frequently (day or night time) was also determined during the preliminary survey. Their estimated numbers were determined by reviewing records of local non-governmental organizations working on street population.

To estimate the number of street people of reproductive age in each selected city, an individual assigned by an authorized person from Women, Children and Youth Affairs, Social and Labor Affairs Office with investigators reviewed non-governmental and governmental organizations’ documents on the street dweller population. In addition, information regarding the number and location of street dwellers in each city were requested from concerned stakeholders (they include the Center of Concern, Elshaday and Development Association Relief and Integrated Service on Health and Development Organization) and a head of Women, Children and Youth Affairs, Justice and Security Office, Zonal Health Department, Social and Labor Affairs Office in each selected city were interviewed by phone.

Finally, 845 samples were allocated proportionally to each selected city. By using an individual who was assigned by an authorized person (from stakeholders/Women, Children and Youth Affairs, Social and Labor Affairs Office) the first study respondents were identified. Then, the snowball sampling technique was used to identify and fill in the required sample size. In the first round, 28 street dwellers were identified. Subsequently, about 30 rounds of snowballing were needed to obtain 845 participants. The study’s respondents who fulfilled the inclusion criteria and volunteered to give information were interviewed. Since participating in the study took time during which they would normally work to support their daily consumption, 50 Ethiopian Birr (approximately 2 US dollars) was given as compensation. Given the cost that participants incurred in getting into the study, this amount was considered adequate but not enough to influence their participation.

Regarding the sample size for the qualitative study, a total of twenty one in-depth interviews and ten informant interviews were conducted. Three in-depth interviews were conducted in each selected city. Respondents for in-depth interviews were selected purposively during the quantitative data collection period. Those respondents who agreed and gave consent to participate in in-depth interviews were appointed a day after the quantitative data collection. One male street dweller with a history of STD and another female street dweller with a history of STD were enrolled from each selected city for in-depth interviews. Furthermore, the key informants’ interviews were conducted by the head of stakeholders such as the Center of Concern, Elshaday and Development Association Relief, Integrated Services on Health and Development Organization, Women, Children and Youth Affairs Office, Justice and Security Office, Zonal Health Department, and Social and Labor Affairs Office. Sectors were randomly selected to have a minimum of one key informant interview in each selected city.

### Data collection tools and procedures

The first sets of data collection tool were pre-tested and structured interviewer-administered questionnaires to collect quantitative data. Relevant literature was reviewed to develop the tool that addresses the objectives of the study [2, 3, 5–8, 10–13]. The pre- test was conducted among 50 street dwellers of reproductive age in non-selected cities. The results of the pre-test were used to modify the instrument. The questionnaire was designed to assess socio-demographic characteristics, and whether respondents had ever heard about infections that can be transmitted through sexual contact, whether they had undergone voluntary counseling and testing, received sexuality education, whether respondents had awareness of prevention and symptoms of STDs and their attitudes towards STDs. Four BSc midwives with previous experience in collecting quantitative and qualitative data were used as data collectors and one MSc holder was employed as a supervisor. To avoid double participation, information regarding the effect of double participation in the quality of data was given to the respondents before data collection proceeded. In addition, the data collectors proceeded to the next city after they completed their duties in each city. To maximize openness of the respondents, male interviewers were assigned for male respondents and female interviewers were assigned for female respondents.

Attitudes towards STDs were measured using six attitudes related questions. A 5-pointLikert scale (with five responses = strongly agree, agree, undecided, disagree, strongly disagree) was used to measure street dwellers attitudes towards STDs. An individual who responded strongly agree for positive attitude was given scores of 5and 1 for those who responded as strongly disagree while the above scores were reversed for negative attitudes questions. Finally the total score (5*6 = 30) was dichotomized into favorable and an unfavorable attitude taking the mean score [15] as a cutoff point. A person with a score at or greater than the mean value of 15 was considered to have a favorable attitude and those with less than 15 were considered to have an unfavorable attitude. Moreover, self-reported STD symptoms and whether they were seeking treatment from health care providers was also assessed. Firstly, they were asked if they experienced symptoms of sexually transmitted diseases such as genital soreness or ulcers, bad-smelling abnormal genital discharge or a burning sensation during urination in the prior year. Secondly, respondents were asked if they sought treatment from health care providers for symptoms. If they did not seek treatment they were asked the reason.

The second set of data collection tools constitutes unstructured questions designed to guide in-depth and key informant interviews. Respondents were informed about the purpose and ultimate use of the information. Then after, permission was obtained from respondents we made an audio recording of the interview. Separate discussion guidelines were used for male and female street dwellers with a history of STD. Furthermore, street dwellers with a history of STD were asked the following questions: Explain what safe sexual practice is. What sexual health problems have you been facing on the street? How do you access treatment options for STDs in your city? “Street on” would intuitively refer to people who are less reliant on the streets of subsistence while “street off” refer to people who are fully reliant on the streets of subsistence and sleep [15].

Stakeholders and service providers were asked questions such as: How do you see the services provided to street dwellers in this sector? What is the responsibility of your sector to solve sexual health problems of street dwellers? What is being done in your city to address the sexual health needs of the street dwellers? How do you approach street people in your cities?

### Data quality control

The quality of the quantitative questionnaire was assured by properly designing and pre-testing the tool, and training the data collectors and supervisors before the actual data collection. Data collectors and supervisors were trained about the aim of the study, procedures, how to approach the study participants and data collection techniques. They were instructed to go through the questionnaire question by question and about how to collect the data. Moreover, clarification was given on the basic skill of communicating with the study participants. Every day after data collection, data were reviewed and checked for completeness, accuracy and clarity by the supervisors and the necessary feedback was offered to data collectors the next morning. Data cleanup and cross-checking were done before analysis. Furthermore, data collection tools were translated into eight local languages in the region.

Similarly, to maintain quality of qualitative data, respondents were informed about the purpose and ultimate use of the information. The most important questions were asked first and the same or parallel questions were asked again for several respondents. The same questions were asked in another way to elicit the same information and they were asked one question at a time. Discussion guidelines were translated into eight local languages in the region to suit the respondents.

### Data management and analysis

The quantitative data were checked manually for completeness, coded and double entered into Epidata version 3.1. After data entry, the data were exported to SPSS version 23.0 for further analysis. Descriptive analysis results are presented in the forms of tables, figures and texts by using frequency distributions and summary statistics such as mean and standard deviation. Both bivariable and multivariable logistic regressionanalyses were used to determine the association of each independent variable with the dependent variable. Variables having *p*-value less than or equal to 0.25 in the bivariable analysis were considered eligible for multivariable logistic regression models. Crude (COR) and adjusted (AOR) odds ratios with their 95% confidence intervals (CIs) were estimated to identify the presence and strength of associations, and statistical significance was declared if *p* < 0.05. Moreover, based on their permission, the voices of the respondents were recorded with a tape recorder. In-depth interview notes were typed and transcribed to the local languages and then subsequently translated to English. Finally, thematic analysis was used as a final model to analyze qualitative data.

## Results

### Socio-demographic characteristics of the respondents

Out of 845 sampled participants 842 responded with a response of 99.6%. More than half 503 (59.7%) of them were male. The mean age of the respondents was 22.9 years (standard deviation (SD) =6.8). Regarding marital status, 521(61.9%) of the respondents were single. In addition, 281 (33.4%) of the street people were enrolled from Hawassa. About two thirds, 550 (65.3%) of the street dwellers current place of residence was different from their original place of residence. Similarly, 568(67.5%) of the respondents were dependent on the street for their life and sleep on the street [street off]. Most 745(88.5%) of them were out of school during the study period. About half 382(45.4%) of street dwellers were living with their peers. Moreover, about two thirds 621(73.8%) of the street dwellers had been living on the street for less than five years (Table 1).

**Table 1:** Socio-demographic characteristics of street dwellers in Southern Ethiopia, 2019 (*n* = 842)

Variables	Frequency	Percent
**Sex**		
Male	503	59.7
Female	339	40.3
**Age in years**		
15–18 years	246	29.3
19–25	407	48.3
≥ 26 years	189	22.4
**Marital status**		
Single/never married	521	61.9
Married	233	27.7
Divorced/separated	73	8.7
Widowed	15	1.8
**Educational status**		
Illiterate	187	22.2
Elementary	542	64.4
High school	102	12.1
College and above	11	1.3
**Schooling status**		
In school	97	11.5
Out of school	745	88.5
**With whom currently living**		
Alone	257	30.5
Peers	382	45.4
Boyfriend/girl friend	135	16.0
Parents	50	5.9
Relatives	18	2.1
**Place of current residence**		
Hawassa	281	33.4
Soddo	165	19.6
Dilla	138	16.4
Arba Minch	117	13.9
Mizan Aman	108	12.8
Yergalem	20	2.4
Durame	13	1.5
**Place of residence prior to starting street life**		
Major urban town	313	37.2
Small town	369	43.8
Rural area	160	19.0
**Type of street**		
Street “on”	274	32.5
Street “off”	568	67.5
**Duration of stay on the Street**		
< 5 years	621	73.8
5–10 years	168	20.0
> 10 years	36	4.3
Could not estimate	17	2.0
**Daily income in birr**		
5–20	136	16.2
21–50	518	61.5
> 51	654	77.7

Regarding the qualitative study, 11 females and 10 males participated in in-depth interviews. About one third, 7(33.33%) of them were from Hawassa. Additionally, 10 key informant interviews with the head of stakeholder organizations such as staff from Women, Children and Youth Affairs, Center of Competence, Elshaday and Development Association Relief, Integrated Service on Health and Development Organization, Justice and Security Office, Zonal Health Department, and Social and Labor Affairs Office were conducted.

### Self-reported symptoms of sexually transmitted disease

About 332 (39.4%) street dwellers reported experiencing symptoms of STDs in the prior year. Among those who experienced symptoms of STD, only 129(15.3%) of them sought treatment from health care providers (Table 2).

**Table 2:** Self-reported symptoms of sexually transmitted disease and treatment sought from health care providers among street dwellers in Southern Ethiopia, 2019 (*n* = 842)

Variables	Frequency	Percent
**Sexually transmitted disease symptoms experienced in the prior year**		
Yes	332	39.4
No	510	60.6
**Sexually transmitted disease symptoms experienced in the prior year**		
Burning sensation during urination	122	14.5
Genital ulcer or sore genitals	94	11.2
Bad-smelling or abnormal genital discharge	116	13.8
**Had sought treatment for symptoms**		
Yes	129	15.3
No	203	24.1
**Reasons for not seeking treatment from health care providers**		
Financial problems	105	12.5
Felt ashamed	100	11.9
The problem was not serious	47	5.6

STDs such as HIV, syphilis and water born disease were also reported in qualitative findings.

A*35 year- old street woman said that*:

“The main problem we have been facing is HIV and syphilis. Offensive vaginal discharge and genital ulcer are the main reason for seeking health care among street dwellers.”

Moreover, the *Center of Concern head nurse stated the following in relation to STDs*:

“Usually they come with different health problems such as unhygienic health condition such as skin disease, and water born disease. That is why we are providing free shower services and educate them on the importance of personal hygiene. The street dwellers are extremely predisposed to STDs; they do not want to tell you. Usually, they tell us that they have difficulty urinating or painful urination. We have an adequate budget to treat them; in severe cases we refer them to Adare General Hospital.”

A 35 year- old street woman from Dilla thought about sexual assault and HIV as follows:

“I have been raped by two persons years ago and I heard that both assaulters died from HIV. I have reported them to the police. One of the assaulters has a wife and his wife went to a district called “Guangua” while he raped me. Anyway, he died from HIV/AIDS. ….…initially I was treated for tuberculosis and finally a health care worker told me that I am a carrier. Now I am using antiretroviral therapy. They told me that my baby is HIV- free, thanks to God.”

### Knowledge of HIV and other STD prevention

Most 780(92.6%) of the respondents had heard about HIV/AIDS. In addition, 299(35.5%) knew that HIV can be transmitted from person to person through unprotected sexual contact and 439(52.1%) of them knew thatHIV can be transmitted by sharing sharp materials with an HIV- infected person. Similarly, 104(12.4%) of the street dwellers knew that HIV can be transmitted from mother to baby during pregnancy, child birth and breast feeding. About two thirds 599(71.1%) of them knew that people can reduce the chance of getting HIV by using a condom every time they have sex. More than half 508(60.3%) of the street dwellers also knew that it is possible for a healthy-looking person to have HIV. The majority, 689(81.8%) of the respondents knew the place where people can go to get an HIV test. Moreover, 569(67.6%) of them had been tested at some point for HIV. Among those who had been tested, 234(27.8%) were tested in the past 3 months, while 257(30.5%) had been tested within the past 6 months and 78(13.7%) of them were tested within the prior year. The major reasons for the most recent test were: 437(51.9%) to learn their HIV status, 54(6.4%) because they had unprotected sexual contact and 97(11.5%) of them were tested to get married.

Most 730(86.7%) of the street dwellers had heard about STDs. In addition, 423(50.2%), 191(22.7%), 48(5.7%), and 68(8.1%) of them knew about HIV, gonorrhea, syphilis and chancroid, respectively. Moreover, 263(31.3%), 271(32.2%) and 176(20.9%) of the respondents knew that genital ulcers, bad-smelling abnormal genital discharge, and experiencing a burning sensation during urination are typical symptoms of STD. Furthermore, 661(78.5%), 261(31.0%), and 268 (31.8%) of the respondents knew that condoms, abstinence and being faithful to one’s partner could prevent STDs. Regarding health education, only about 219(26.0%) of the respondents received any health education about STDs in the previous year. In the previous year 114(17.1%) of the respondents visited health institutions to get information or treatment about STDs.

Moreover, the findings of in-depth interviews with street dwellers also raised subsequent ideas regarding knowledge about unsafe sexual practice and its consequences:

A *38 year- old street woman said that:*

*“*I do not know what unsafe sexual practices mean; it might be any sexual act that exposes someone to HIV and syphilis.”

Moreover, *an 18 year-old street male also added the following points:*

“The consequences of sexual intercourse I fear are pregnancy only. It is a must to use a condom if she is HIV positive unless there is no need to use a condom. I do not think that having multiple sexual partners is a problem. It is normal for us.”

Similarly, an *18 year-old street male also said that:*

“If you have sexual desire it is a must to have sexual contact with your neighbor street friends. I do not know what types of STD I can acquire.”

A *24 year-old street male also mentioned the following:*

“Due to different reasons the women joins street life; then they had intercourse with nearby street friends and gave births. Now, their baby will grow up on the street and become a street boy/girl. You can see a number of street females having a child in front of the bus station and church.”

### Attitude towards sexually transmitted disease

About half 393 (46.7%) of the respondents disagreed with idea that most STDs (except HIV) can resolve by themselves without any treatment. Moreover, most 711(84.4%) of them had a favourable attitude towards STDs (Table 3).

**Table 3:** The extent to which participants agreed or disagreed with statements about their attitudes towards sexually transmitted disease among street dwellers, Southern Ethiopia, 2019

Variables	Strongly agree	Agree	Neutral	Disagree	Strongly disagree
	N (%)	N (%)	N (%)	N (%)	N (%)
It is possible for street dwellers to become infected with STD from a single intercourse	63 (7.5)	128 (15.2)	102 (12.1)	311 (36.9)	238 (28.2)
Most STDs (except HIV) are not very serious	68 (8.1)	230 (27.3)	231 (27.4)	279 (33.1)	34 (4.0)
Most STDs (except HIV) can resolve by themselves without treatment	43 (5.1)	144 (17.1)	167 (19.8)	393 (46.7)	95 (11.3)
All contraceptive methods can prevent STDs	30 (3.6)	185 (22.0)	275 (32.7)	288 (34.2)	64 (7.6)
Condom use can prevent STDs	36 (4.3)	141 (16.7)	142 (16.9)	351 (41.7)	172 (20.5)
A Woman should ask her sexual partner to use a condom if she wants him to	66 (7.8)	359 (42.6)	214 (25.4)	158 (18.8)	45 (5.4)

In bivariate analysis, the factors found to be significantly associated with attitude towards STDs were sex, age, type of street, place of current residence, current place of residence different from original place of residence, place of residence prior to joining street life, and been previously tested for HIV. From variables found to be significant in bivariate analysis, age, place of current residence, place of residence prior to joining street life and were previously tested for HIV were found to be significantly associated with attitude towards STDs in multivariable logistic regression analysis.

Thus, those aged 19–25, and 26 years and above were about 2 [AOR: 2.24; CI: 1.23, 4.09] and 5 times [AOR: 4.67; CI: 2.37, 9.15] more likely to have a favourable attitude towards STDs compared to those who were 15–18 years, respectively. Similarly, currently living in Soddo and Dilla towns was associated with a greater likelihood of having favourable attitude towards STDs compared to those living in Hawassa town [AOR: 12.19, CI: 6.43, 23.12], AOR: 4.48, CI: 2.14, 9.35], respectively. In addition, the place of residence prior to joining street life was also significantly associated with attitude towards STDs. Those who were living in small towns prior to joining street life were about 3 times more likely to have a favourable attitude towards STDs compared to those who were living in bigger cities [AOR: 2.88; CI: 1.44, 5.77]. Furthermore, those who were previously tested for HIV were about 2 times more likely to have a favourable attitude towards STDs compared to those who were never tested [AOR: 1.68; CI: 1.05, 2.67] (Table 4).

**Table 4:** Factors associated with attitudes of respondents towards STDs among street dwellers in Southern Ethiopia (Bivariate and Multivariable Analyses), 2019 (*n* = 842)

Variables	OR (95% CI)	
	COR(95% CI)	AOR(95% CI)
**Sex**		
Male	0.425 (0.28, 0.65)	*
Female	1	
**Age in years**		
15–18	1	
19–25	1.65 (1.01, 2.69)	**2.24 (1.23,4.09)**
26 and more	2.53 (1.48, 4.32)	**4.67 (2.37,9.15)**
**Current place of residence**		
Hawassa	1	
Soddo	16.56 (9.05,30.33)	**12.19 (6.43, 23.12)**
Dilla	3.24 (1.69,6.22)	**4.48 (2.14, 9.35)**
Arba Minch	1.38 (0.17,11.29)	0.58 (0.07, 5.12)
Mizan Aman	1.51 (0.64,3.52)	1.19 (0.48,2.93)
Yergalem	1.05 (0.42,2.63)	0.58 (0.21,1.57)
Durame	1.84 (0.39,8.63)	2.28 (0.46,11.41)
**Current place of residence is different from original residence**		
Yes	1.34 (0.72, 1.97)	*
No	1	
**Place of residence prior to joining street life**		
Major urban town	1.23 (0.68,2.25)	1.51 (0.67, 3.38)
Small town	2.11 (1.20,3.70)	**2.88 (1.44,5.77)**
Rural area	1	
**Type of street**		
Street on	0.64 (0.44,0.94)	*
Street off	1	
**Ever tested for HIV**		
Yes	2.47 (1.69,3.61)	**1.68 (1.05, 2.67)**
No	1	

* = Not significant in backward stepwise logistic regression

### Health care service accessibility and challenges

Both in-depth interviews with street dwellers and key informants’ interviews with stakeholders corroborate that unavailability of the services and lack of special facilities stands in the way of serving street dwellers.

In relationship to this, a 24 year- old young street male stated the following points:

“Reproductive health services are not accessible for street people. That is why street dwellers are afraidto go to a health facility because we fear questions raised by providers. Health care providers do not want to care for street dwellers and they believe that all of us are prostitutes, aimless and thieves.”

Adding to this point, 25 year-old street female said:

“Accessibility of the condom by itself is an extra problem; it is not easy to buy condoms for “Borko” to mean *extremely poor and jobless.*”

Another, *27 year-old street male also added the following points:*

“When we contract STDs, usually we go to a clinic and take tablets. Accessibility to the service is similar to other people. There is no special facility that serves street dwellers.”

Moreover, a 19 year-old street male stated as follows:

“To address street dwellers’ sexual and reproductive health needs, we expect the government should teach us about HIV, family planning and effect of unwanted pregnancy on the life of street dwellers.”

A representative from the Social and Labor Affairs office also added the following points regarding the awareness of street dwellers about HIV and STDs:

“Most street dwellers come from rural areas and they are illiterate. They usually came to the city for looking for a good living condition. They do not have adequate information about HIV and other STDs, how to live and get jobs in the city. This caused them to join street life. They speak to you as if they know about HIV, but if you asked them about mode of transmission and prevention mechanism they do not know.”

Besides, another *Social and Labor Affairs Offices employee also added the following points regarding to the accessibility of sexual health education:*

“The Health office should provide continuous awareness creation about risky sexual behavior, HIV and STDs. I have seen some youths that have no skill about how to use a condom.”

In addition to this, the *Integrated Service on Health and Development Organization head of staff also included the following points:*

“Every customer [street dwellers and commercial sex workers] that visits our organization will receive the option for voluntary HIV counseling and testing, and will be screened for STD and treated free of charge. If we diagnose HIV, we initiate ART drug therapy here. There are around 3,500 commercial sex workers in Hawassa. Every day, around 30-40 commercial sex workers visit our office and every service is documented. Their sexual partners might be drivers, street dwellers and daily laborers.”

Further, another *29 year-old street woman also adds the following points:*

“We are being exposed to different reproductive health problems due to a lack of protection and home. Lack of knowledge about STDs, condom use and family planning is our problem. For instance, I heard about HIV 3 years back while, I gave birth my last baby at the health center. Then after; no one thought us about HIV, family planning and STD.”

## Discussion

This study assessed knowledge, attitude and STDs treatment-seeking behaviors of street dwellers in southern Ethiopia. Even though, most 730(86.7%) of street dwellers had heard about STDs, only 114(17.1%) of them visited a health institution to get information about or treatment for STDs in the previous year. This is also supported by the qualitative findings of the current study. This might indicate that street dwellers are marginalized and not getting timely information about their sexual or reproductive health due lack of information on available services [16].

In the present study, most 711(84.4%) of the street dwellers had a favourable attitude towards STDs. Moreover, about half 393(46.7%) of the respondents disagreed with the idea the most STDs (except HIV) can resolve by themselves without any treatment. This finding is higher than that reported from a study conducted in the Kathmandu Valley [18]. This might be due to a difference in study periods and procedures. For instance, the study in Kathmandu Valley was conducted in 2004, which is difficult to compare with the present attitudes under the influence of a range of technologies. In this study, the age of the respondents was significantly associated with attitudes towards STDs. Those in the age range of 19–25 years and 26 years and above were more likely to have a favourable attitude towards STDs compared to those who were 15–18 years. As the age of the respondents increased, there was greater likelihood of having a favourable attitude towards STDs. This is possibly explained by a greater chance of attending a health care facility, receiving health education and sharing information about STDs with peers at an older age. That is why a previous study indicated the vulnerability of young age for HIV infection [6].

In addition, those who were living in Soddo and Dilla towns had a greater likelihood of having a favourable attitude towards STDs compared to those who were living in Hawassa city. This might be because of the size of the towns and relatively manageable number of street dwellers in Soddo and Dilla towns which made it easier for them to attend prevention and social support programs [6]. Since Hawassa is the capital city of SNNPRS, the number of street dwellers is expected to be much greater than the number of street dwellers in Soddo and Dilla [14]. Hence, those who were living in Soddo and Dilla towns might more easily access sexual health education conducted by stakeholders that could consequently improve their attitude towards STDs than those were living in Hawassa. In addition, participants’ place of residence prior to joining street life was also significantly associated with their attitude towards STDs. Those who were living in small towns prior to joining street life were more likely to have favourable attitude towards STDs compared to those who were living in major cities. This could be due the fact that those who were from small towns might be more concerned about STDs because of the challenge they faced while relocating to bigger cities. Moreover, those who were previously tested for HIV were about two times more likely to have a favourable attitude towards STDs compared to those who were never tested. The reason for this is likely the information gained during pre and post test counselling.

More than one third 332(39.5%) of the street dwellers did experience at least one symptom of STD in the previous year. This finding is in line with a study conducted in Brazil (39.6%) [6]. Similarly, a 35 year-old woman from Dilla also said thatthe main problem street females have been facing is HIV and syphilis. The present finding is higher than the narrative review in US (6 to 32%) [5]. The possible explanations for this difference could be differences in socio-demographic characteristics, methodology and time period of the study. For instance, the narrative reviews of US consider articles from 2000 to 2015.

About two thirds, 203(61.14%) of participants had not sought treatment from health care providers for self-reported symptoms. This might be due to the fact that street dwellers had a greater likelihood of substance use, exchanging sex for money and risky sexual practices which in turn increased self-reported STDs [8]. The possible reason for not seeking treatment might be due to perceived discrimination by health care providers, fear over confidentiality and lack of accessibility to treatment centers [9]. Moreover, a 27 year-old street male from Wolaita Soddo pointed out that accessibility of health services is similar to other people. Similarly, a 25 year-old street female from Hawassa said as follows; there is no special facility that serves street dwellers. Accessibility of the condom is another problem; it is not an easy to buy condoms for “Borko” (extremely poor and jobless).

### Limitations of the study

Since this study assessed STDs among street dwellers, as in all self-reported studies, we cannot rule out social desirability bias to sensitive questions, which might have introduced biases of unknown magnitude and direction though the study was anonymous and we arranged for same-sex interviewers.

## Conclusions

In this study, a significant number of street dwellers experienced self-reported symptoms of STDs in the prior year. In addition, despite having an awareness about STDs, treatment seeking behaviors of street dwellers from health care facilities were found to be low as established by both quantitative and qualitative findings. Moreover, a considerable percentage of street dwellers in the present study had a favorable attitude towards STDs. Further, age, place of current residence, place of residence prior to joining street life and being previously tested for HIVwere factors found to be significantly associated with attitudes towards STDs. We recommend that health care providers be provided with special training to address sexual and reproductive health problems of street dwellers. The Ministry of Health and other stakeholders should work to improve the knowledge and attitudes of street dwellers about STDs.

## Data Availability

All the datasets used and analyzed during the current study are available from the manuscript.

## Abbreviations

STDs: Sexually Transmitted Diseases
SNNPRS: Southern Nation, Nationality and People Regional State
